# Structure of Signaling Enzyme Reveals How Calcium Turns It On

**DOI:** 10.1371/journal.pbio.1000427

**Published:** 2010-07-27

**Authors:** Richard Robinson

**Affiliations:** Freelance Science Writer, Sherborn, Massachusetts, United States of America

**Figure pbio-1000427-g001:**
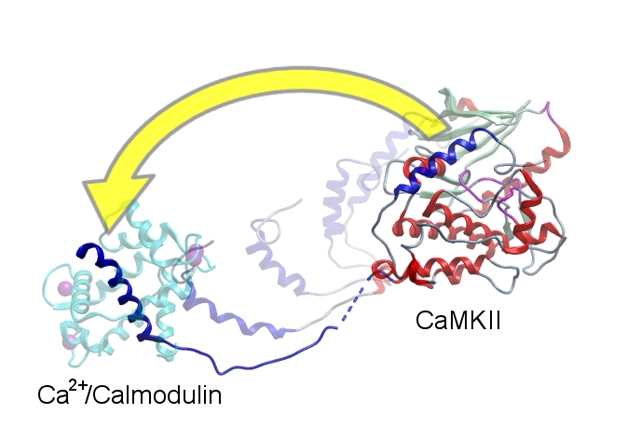
Inactive CaMKII (right) becomes activated when the inhibitory domain (blue helix) is dislodged from the active site (left), after passing through multiple intermediate states.

[Fig pbio-1000427-g001]Calcium signaling is central to many of the most important cellular processes, from intracellular transport to memory. In most cases, calcium first joins up with a protein intermediary, calmodulin. With calcium on board, calmodulin (now called Ca^2+^/CaM) can bind to a large variety of cellular targets, altering their structure and thus their function.

Calcium is a key signaling molecule, its levels under tight regulatory control in cells. In neurons, long-lasting contact between synapses (also called long-term potentiation) increases calcium levels above a critical threshold. Though extremely short-lived, these calcium spikes lead to the activation of the enzyme calcium/calmodulin-dependent kinase II (CaMKII). Unlike many other Ca^2+^/CaM targets, CaMKII can remain active long after the local calcium spike that activated it has subsided. As a result, CaMKII functions as a molecular switch that activates downstream signaling processes that in turn modulate synaptic strength, the molecular basis of memory.

Despite the importance of CaMKII activation, a complete understanding of the mechanism underlying it has been elusive, largely because the structure of the catalytic and regulatory domains in complex with Ca^2+^/CaM has not been resolved. In this issue of *PLoS Biology*, Peter Rellos, Stefan Knapp, and colleagues determine the enzyme's structure in its activated form, revealing the molecular events that switch on catalytic CaMKII activity in the presence of calcium.

CaMKII is a kinase, meaning it adds phosphates to other molecules, altering the structure of the target molecule. In common with many other enzymes, the active site of CaMKII is a groove on the protein's surface. It had previously been shown that in the inactive form of the enzyme, the groove is occupied by a helical inhibitory domain located near the catalytic domain; the helix blocks entry of the substrate, thus preventing phosphorylation of the target. It was also known that multiple copies of CaMKII can link up to form a large oligomeric structure, and that the helix of one CaMKII is a target for phosphorylation by an adjacent CaMKII. Once phosphorylated, the helix no longer fits into the groove, and the enzyme therefore becomes active—this is the structural basis for the prolonged activation of the enzyme. But how is the helix dislodged in the first place?

To answer that, the authors crystallized the portion of the protein containing the active site and the helix, along with calcium/calmodulin in its binding site, thus capturing major features of the activated form of the enzyme. They found that Ca^2+^/CaM binding induced conformational changes in both the active site and the inhibitory helical regions. The inhibitory helix was dislodged from the pocket on its own enzyme and was found instead in close proximity to the (now open) substrate pocket of an adjacent kinase. This suggests that the adjacent kinase could then phosphorylate the helix, preventing the helix from re-lodging in the pocket of its own kinase.

While suggestive, the X-ray crystallograph is a snapshot—not a movie—and doesn't by itself prove that the adjacent kinase actually engages the helix. But the authors also showed, using analytical ultracentrifugation, that the two copies of the enzyme associated in the presence of Ca^2+^/CaM and that association could be disrupted when the active site was blocked by the inhibitory domain, confirming its role in linking the two enzymes.

In vivo, 12 molecules of CaMKII combine to form a multi-enzyme complex, a dodecamer built from two six-member rings stacked one atop the other. Combining their crystallographic structures of the various parts of the whole, the authors constructed a convincing model of the holoenzyme. In the model, each active site on one ring is paired up with an active site on the other ring. Binding of Ca^2+^/CaM molecules, one per CaMKII, bumps the inhibitory helices out of their own active sites and across the ring to the adjacent ones. At the same time, their structure showed, binding of Ca^2+^/CaM repositions a surface amino acid on each CaMKII so that it can better grab ATP, which the active site uses as a phosphate source, increasing the efficiency of the enzyme. With the active site open, the helices in place, and phosphate at hand, the enzymes phosphorylate the helices, disabling them from reblocking the active site. Thus, the enzyme remains active even after the calcium concentration returns to normal.

Because aberrant CaMKII activity has been implicated in human disease, these results are likely to provide a rational basis for drug development aimed directly at the enzyme's mechanism. While targeting such a centrally important protein runs the risk of altering too many cell processes, the cell employs multiple forms of the enzyme, and it may be possible to use their subtle structural differences to manipulate the activity of one while leaving the others largely alone.

**Rellos P, Pike ACW, Niesen FH, Salah E, Lee WH, et al. (2010) Structure of the CaMKIIδ/Calmodulin Complex Reveals the Molecular Mechanism of CaMKII Kinase Activation. doi:10.1371/journal/pbio.1000426**

